# Kangyi Qiangshen Gong exercise prescription for pulmonary function and quality of life in patients recovered from COVID-19: a study protocol for a randomized controlled trial

**DOI:** 10.1186/s13063-022-06817-5

**Published:** 2022-10-14

**Authors:** Guangxin Guo, Xiruo Xu, Wong Yu Yin, Kunyu Zhang, Jacelyn Pang Min Hui, Janice Hiew Yuen Yee, Bryan Chung Qi Heng, Yuan Qin, Fei Yao, Min Fang

**Affiliations:** 1 Shanghai Municipal Hospital of Traditional Chinese Medicine, Shanghai University of Traditional Chinese Medicine, Shanghai, 200071 China; 2 School of Acupuncture-Moxibustion and Tuina, Shanghai University of Traditional Chinese Medicine, Shanghai, 201203 China; 3 International Education College, Shanghai University of Traditional Chinese Medicine, Shanghai, 201203 China; 4 School of Pharmacy, Shanghai University of Traditional Chinese Medicine, Shanghai, 201203 China; 5 Yueyang Hospital of Integrated Traditional Chinese and Western Medicine, Shanghai University of Traditional Chinese Medicine, Shanghai, 200437 China

**Keywords:** COVID-19, Qigong, Traditional Chinese medicine rehabilitation, Randomized controlled trial, Study protocol

## Abstract

**Background:**

Since early 2022, patients with 2019 novel coronavirus (COVID-19) infection have increased rapidly in Shanghai, China. Nevertheless, there is no widely used unified rehabilitation treatment available for discharged patients with post-infection sequelae such as dyspnea, depression, and fatigue. To promote the rehabilitation of discharged patients, our team formulated Kangyi Qiangshen Gong exercise prescription on the basis of traditional Chinese medicine rehabilitation exercises (TCMRE). We designed a randomized controlled trial to evaluate the efficacy of rehabilitation and advantages of KQG for discharged patients with post-COVID-19 syndrome.

**Methods/design:**

This is a parallel-design, two-arm, analyst assessor-blinded, randomized controlled trial. In total, 60 discharged patients with COVID-19 sequelae, aged from 20 to 80 years will be recruited and randomly assigned to the World Health Organization instructed breathing techniques (BT) group and the Kangyi Qiangshen Gong exercise prescription (KQG) group at a ratio of 1:1. The patients in the BT group will perform breathing techniques exercise, and the patients in the KQG group will perform KQG exercise. Both groups will perform exercises twice a day for 3 months. The primary outcome will be measured with the Modified Medical Research Council Dyspnea Scale, and the secondary outcomes will include the Modified Borg Scale, Fatigue Scale-14, Patient Health Questionnaire-9 Scale, Pittsburgh Sleep Quality Index, and the Respiratory Symptoms Scale. Clinical scales will be assessed at three points (pre-exercise, 3 months post-exercise, and 3 months follow-up). Adverse events will be recorded for safety assessment.

**Discussion:**

This trial will serve high-quality evidence of the value of KQG for treating discharged patients with COVID-19 in rehabilitation period.

**Trial registration:**

Chinese Clinical Trial Registry ChiCTR2200059504. Registered on 03 May 2022.

**Dissemination:**

The results will be published in peer-reviewed journals and disseminated through the study’s website, and conferences.

## Background

COVID-19 epidemic is still expanding at a global level. Across the six World Health Organization (WHO) regions, over 7 million new cases and over 26,000 new deaths were reported. As of 12 April 2022, over 496 million confirmed cases and over 6 million deaths have been reported globally [1]. In March 2022, the number of patients with COVID-19 increased rapidly in Shanghai, China. As of April 12, 2022, 9903 patients had been diagnosed with COVID-19 infection in Shanghai, and 224,704 asymptomatic infections were still under medical observation [2]. With the continuous help of other provinces in China, the number of discharged COVID-19 patients in Shanghai gradually increased. The natural course of COVID-19 remains ill-defined, such as the frequency, nature, and duration of persistent symptoms, which presents the knowledge gap of the doctors [3]. Most previous studies have focused on hospitalized patients with COVID-19 rather than the sequelae of discharged patients. But it is reported that COVID-19 convalescent patients may leave sequelae, including dyspnea, fatigue, depression, sleep problems, or cognitive impairment (brain fog) [4–7]. Post-COVID-19 syndrome deteriorates the quality of life of discharged patients with COVID-19 [8, 9]. There is an urgent need to develop therapeutic strategies for effective rehabilitation of discharged COVID-19 convalescent patients [7, 10].

Prior studies [11–13] demonstrated that guidance on symptom management of COVID-19 patients in physical training had often been derived from clinical experiences and guidelines for the treatment of patients with other illnesses. Evidence on the efficacy and safety of palliative symptom control in COVID-19 patients remains lacking [13]. However, a recent study showed that exercises are beneficial to the rehabilitation of patients with post-COVID-19 syndrome by directly enhancing the respiratory muscle strength and the ability of pulmonary ventilation, inhibiting the storm of inflammatory cytokines, inhibiting intracellular and external oxidative stress, and regulating the intestinal flora [14]. Evidence suggests positive long-term effects of respiratory physiotherapy in post-acute COVID-19 adult patients [15].

Traditional Chinese exercise is a popular physical exercise in China. A systematic review showed that alternative medicine (CAM) like Qigong can significantly improve various psychological symptoms and physical symptoms in COVID-19 patients [16]. Previous studies [17] have shown that KQG is suitable for in-patients with mild or moderate COVID-19, which can significantly improve the physical quality of patients, enhance cardiopulmonary function, smooth emotions, and reduce fatigue. In China, only a rapid guide for exercise rehabilitation of discharged COVID-19 patients is provided, including traditional Chinese exercise rehabilitation methods such as Liu Zi Jue Qigong, Baduanjin Qigong, and Taijiquan [18–21]. Qigong is a traditional Chinese mind–body exercise and can improve sleep problems, fatigue symptom clusters, and depression [22–24]. In order to improve COVID-19 patients’ cardiopulmonary function, emotional disorder, and quality of life, we developed Kangyi Qiangshen Gong exercise prescription on the basis of previous researches [17, 25] and according to the application of multiple hospitals in Wuhan, China. KQG is a further optimized on the basis of Liuzijue Qigong, Baduanjin Qigong, Yijinjing Qigong, Wuqinxi, and Tai Chi.

However, there is still a lack of clinical evidence for its efficiency in the sequelae of discharged COVID-19 patients. We believe that KQG is also applicable to rehabilitation patients who meet the discharge criteria and can be used as an effective TCMRE therapy for family-centered intervention in the future. KQG has shown potential clinical effect on the various dimensions of COVID-19 which may improve patients’ quality of life, help them get back to work, and thus reduce the healthcare and economic burden on the society. This study can also provide evidence of evidence-based medicine on the KQG treatment of post-COVID-19 sequelae, which will contribute to the development of international expert consensus and play an important role in the prevention and treatment of the epidemic through traditional Chinese physiotherapy. Therefore, we designed a randomized controlled trial (RCT) to evaluate the rehabilitation efficacy and advantages of KQG in the treatment of discharged patients with post-COVID-19 sequelae. We hypothesize that Kangyi Qiangshen Gong exercise prescription will be more effective than breathing techniques in improving COVID-19 sequelae, which can be used as an effective TCMRE therapy for family-centered intervention in the future.

## Methods/design

### Study design

**Fig. 1:**
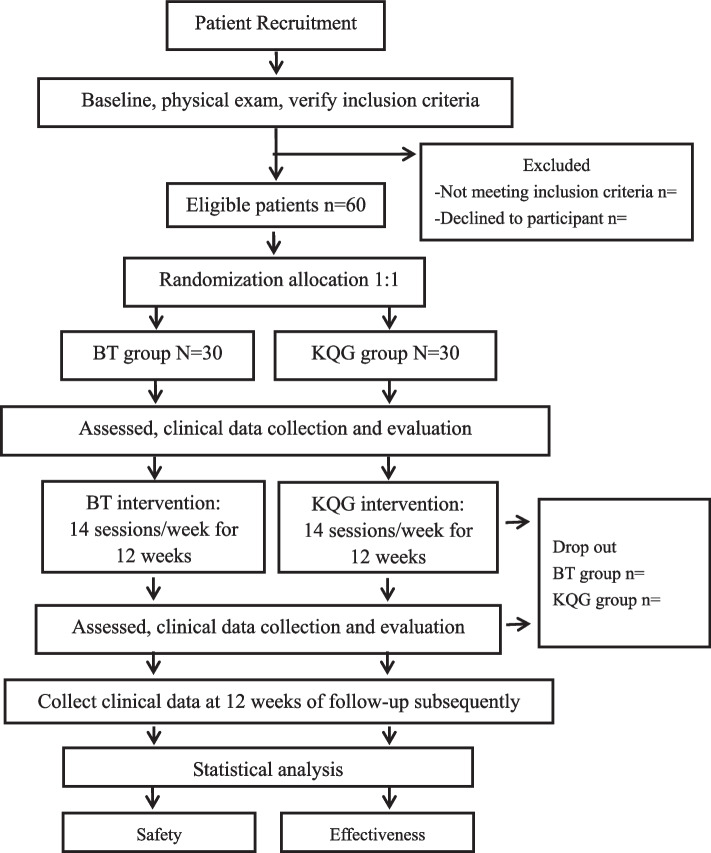
Flow chart of the trial. The present study is a randomized controlled trial. Sixty patients will be included and randomized equally to two groups, a BT group and a KQG group. For 30 patients in each group, this trial will include a 12-week treatment period. During the treatment, patients in the BT group will receive breathing techniques intervention, while the KQG group will receive KQG intervention. Both the outcome assessments will be performed at 3 time points, namely the baseline, the end of the treatments at 12 weeks, and 12 weeks of follow-up subsequently

**Fig. 2:**
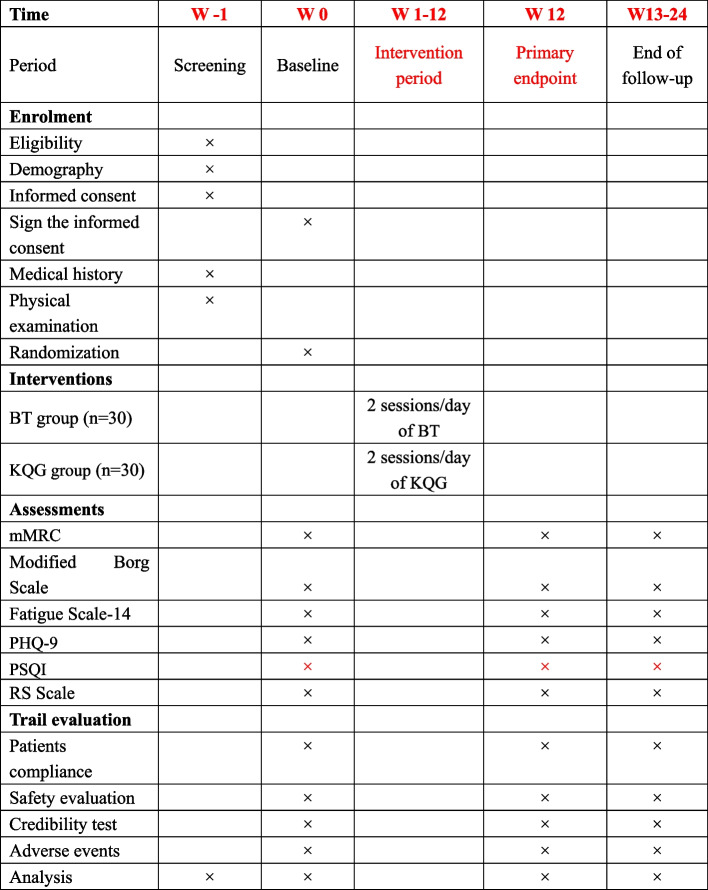
Study schedule showing the time points for enrollment and assessment. ADL, activities of daily living; CRF, case report form; CT, computed tomographic; mMRC, modified Medical Research Council; PHQ-9, Patient Health Questionnaire-9; PSQI: Pittsburgh Sleep Quality Index; RS, respiratory symptoms; TCMR, traditional Chinese medicine rehabilitation

This will be a single-center, parallel-arm, superiority randomized controlled trial. A total of 60 patients will be recruited from the Shanghai Municipal Hospital of Traditional Chinese Medicine, Shanghai University of Traditional Chinese Medicine. All patients will be provided written informed consent at the time of recruitment. Patients with symptoms of dyspnea will be recruited and will have equal opportunity to be randomly assigned to either the BT group or KQG group. Only outcome assessors and statisticians will be blinded due to the limitations of the intervention methodology. Outcomes will be assessed and analyzed by the assessors at three time points (pre-intervention, 3 months post-intervention, and 3 months follow-up). Data management and statistics will be conducted at the School of Acupuncture-Moxibustion and Tuina, Shanghai University of Traditional Chinese Medicine (SUTCM). The flowchart in Fig. 1 illustrates the study design, and Fig. 2 shows the study schedule. Additional file 1 shows the Standard Protocol Items: Recommendations for Interventional Trials (SPIRIT) checklist.

### Participant recruitment

Patients recovered from COVID-19 formulated by post-COVID-19 symptoms will be recruited. In our study, subjects will be recruited from Shanghai Municipal Hospital of Traditional Chinese Medicine, Shanghai University of TCM. Recruitment methods included WeChat, online advertisements, posters, and leaflets. WeChat has currently been regarded as the most popular instant-messaging platform in China, with a large population base. Both the medical staff and patients have their own WeChat account, which make it easy and efficient to communicate online. Shanghai Municipal Hospital of Traditional Chinese Medicine is a tertiary hospital among the designated hospitals for the treatment of COVID-19 in Shanghai. Shanghai Municipal Hospital of Traditional Chinese Medicine has set up a specialized COVID-19 Rehabilitation Clinic for patients recovering from severe COVID-19 to provide TCM rehabilitation consultation, facilitating home rehabilitation through platforms such as internet hospitals, which could be a potential source of subjects. Formal screening process for participants will be conducted by the principal investigators (LF) or study coordinator (WC) to ensure the fulfillment of criteria below and informed consent form.

### Inclusion criteria

Patients with the following criteria will be recruited:

(1) discharged patients with COVID-19; (2) are male or female between the ages of 20 and 80; (3) have post-COVID-19 symptoms include but not limited to breath difficulties, fatigue, sleep difficulties, anxiety, or depression; (4) have a stable condition and remain conscious and cooperative during trial; (5) willing to join the trial and signed the informed consent form; (6) promise not to participate in other forms of exercise.

### Exclusion criteria

Any involvement in the following condition will be removed: (1) patients with complications of underlying noncommunicable diseases such as chronic obstructive pulmonary disease, coronary heart disease, dementia, chronic kidney disease, immunosuppression, and cancer; (2) patients with mental illness; (3) patients with cognitive dysfunction until unable to understand the whole trial process; (4) patients with severe bone or joint diseases that affect movement (e.g., spinal arthritis, severe osteoporosis, and periarthritis); (5) patients with pregnancy or lactating; (6) cigarettes smokers; (7) patients who participate in other form of exercise programs during trial.

### Dropout and suspension criteria

Under the protection of the Declaration of Helsinki [26], patients are allowed to withdraw themselves from the clinical research at any time. In addition, if the intervention time is below 3 months, subjects will be considered as not completed the outcome evaluation.

### Interventions

In this study, a total of 60 recruited participants will be randomized into the BT group or the KQG group. Both exercises last 15 min per time and each program will be performed twice a day at 10 am and 4 pm [27]. Thus, a total of 30 min will be needed every day [28]. A 3-week treatment comprised one course. The intervention will last for 3 months in both our groups and then both will be followed up for a further 3 months.

The exercise of both groups will be demonstrated and monitored by the therapists on the first day until the patient is able to perform the exercises proficiently. Text instruction and video demonstration of the exercises will be provided to patients for subsequent practice. After patients are familiar with the basic movements, they can increase the intensity and focus on the breathing under the guidance of the therapists. Therapists performing TCMRE will be required to have a minimum of 10 years’ clinical experience in Qigong therapy, and they must have undergone meticulous clinical trial training before conducting trials until passing an exam to perform the intervention. In addition, participants in both groups will be allowed to exercise normally under intervention conditions.

The exercise of both groups will be demonstrated and monitored by the therapists on the first day until the patient is able to perform the exercises proficiently. Paper and video versions of the exercises will be provided to patients for subsequent practice. After patients are familiar with the basic exercise, they can increase the intensity and focus on the breathing under the guidance of the therapist. Therapists performing TCMRE will be required to have a minimum of 10 years clinical experience in Qigong therapy. And they must have undergone meticulous clinical trial training before conducting trials until passing an exam to perform the intervention. In addition, participants in both groups will be allowed to exercise normally under intervention conditions.

#### Breathing techniques group (BG)

Breathing technique (BT) is a self-management device in strict accordance with *WHO Europe, Support for Rehabilitation: Self-management After COVID-19-Related illness, Second Edition*. The leaflet was written by rehabilitation professionals in consultation with people recovering from COVID-19. For adults who are recovering from COVID-19, breathing technique can be used by individuals after hospitalization from the illness and those in the community who did not need hospitalization [29].

**Fig. 3:**
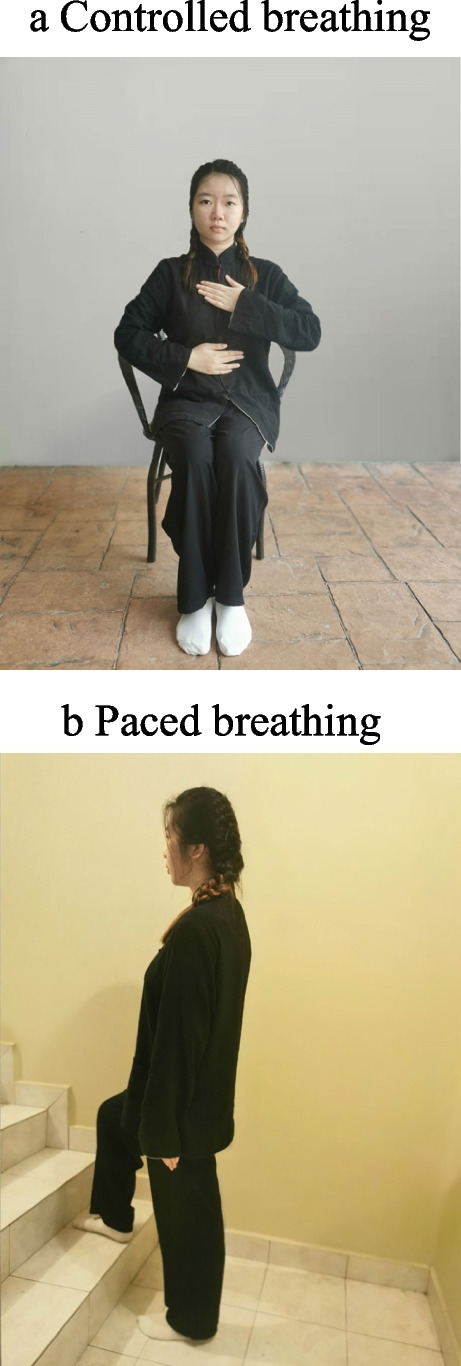
Breathing techniques group (BG). **a** Controlled breathing helps in relaxing and controlling breathing. Patient is required to sit in the position with one hand on chest and the other on stomach. Relax and focus on breathing. Inhale slowly and smoothly through nose, exhale through mouth. The hand on stomach should rise more than the hand on chest. **b** Paced breathing breaks the activity down into smaller parts. Inhale before making the effort of the activity. Exhale when making the effort of the activity. Note: this technique is carried out with activities that require more effort, such as climbing the stairs or walking up the hill

Breathing techniques exercises are performed as follows (Fig. 3).

- Controlled breathing helps in relaxing and controlling breathing. Patient is required to sit in the position with one hand on chest and the other on stomach. Relax and focus on breathing. Inhale slowly and smoothly through nose, exhale through mouth. The hand on stomach should rise more than the hand on chest.
- Paced breathing breaks the activity down into smaller parts. Inhale before making the effort of the activity. Exhale when making the effort of the activity. Note: this technique is carried out with activities that require more effort, such as climbing the stairs or walking up the hill.

#### Kangyi Qiangshen Gong group (KQG)

KQG is the combination of Taiji, Yijinjing Qigong, Shaolin Neigong, Wuqinxi Qigong, Liuzijue Qigong, and Baduanjin Qigong. In conformity with the standard therapy, the KQG group will perform a standardized TCMR program of KQG which consists of seven sets of exercises in total [28].

**Fig. 4:**
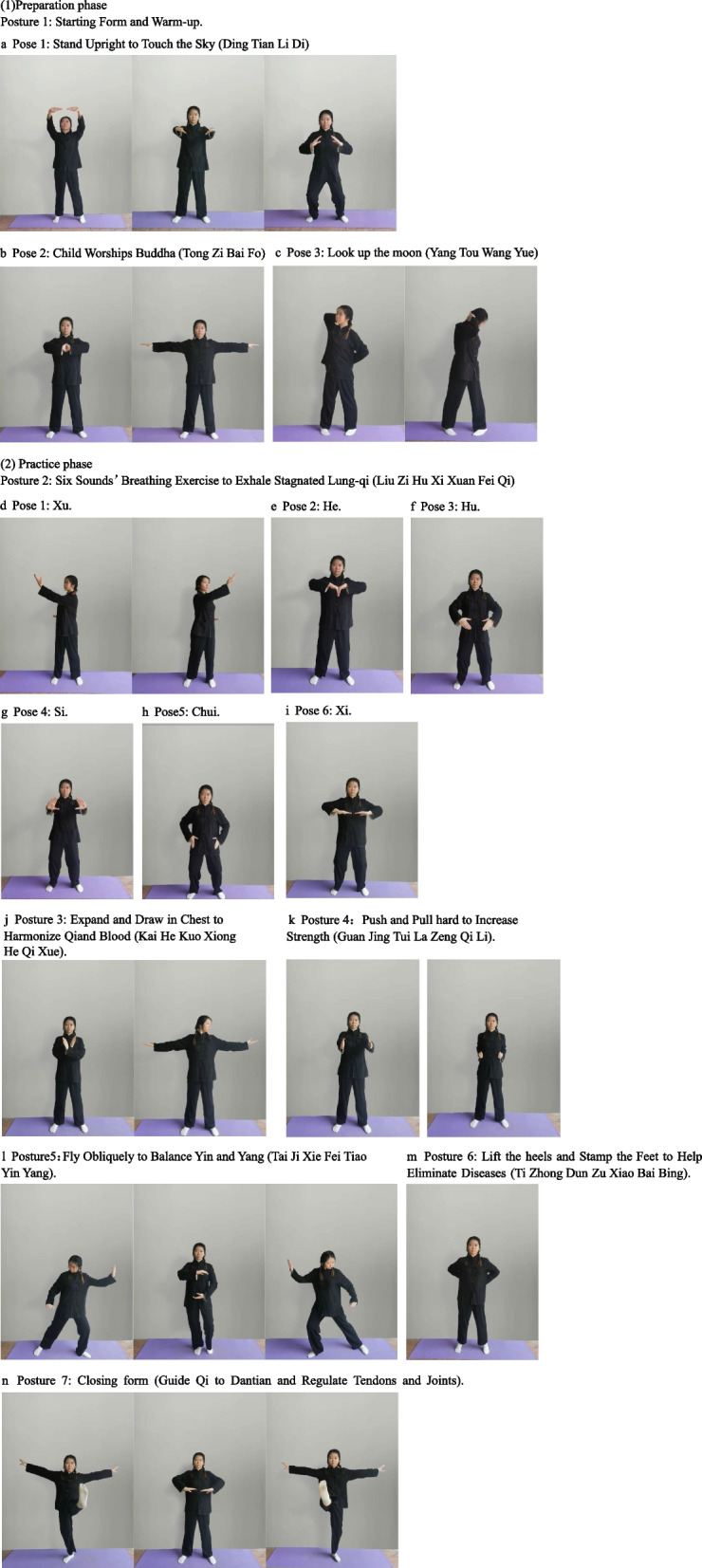
Kangyi Qiangshen Gong group (KQG). (1) Preparation phase. Posture 1: Starting form and warm-up. This posture consists of three poses including Ding Tian Li Di (Stand upright to touch the sky), Tong Zi Bai Fo (Child Worships Buddha), Yang Tou Wang Yue (Looking up to the moon). **a** Pose 1: Stand upright to touch the sky (Ding Tian Li Di) Lift the hands slowly, press down. Raise hands in front of the chest, with knees slightly bent. Lift hands above the head, with palms facing up, fingertips facing, while standing on your toes. Stretch upward, hold for 5 s, return to standing position. **b** Pose 2: Child worships Buddha (Tong Zi Bai Fo). Arms raised from the sides of body, parallel to the floor, and stretch outwards, then hold for 5 s. Press palms together, flip hands 180°, and align the fingertips towards Dan Zhong acupoint, breathe naturally, hold for 5 s. **c** Pose 3: Look up the moon (Yang Tou Wang Yue). Turn toes inward, support the back of neck with the right hand, and the back with the left hand. Raise the head to the right 45° and twist waist, hold for 5 s then return. Twist waist and look at the left heel, hold for 5 s. Support the back of neck with the left hand, and the back with the right hand. Raise the head to the right 45° and twist waist, hold for 5 s then return. Twist waist and look at the right heel, hold for 5 s. (2) Practice phase. Posture 2: Six sounds’ breathing exercise to exhale stagnated lung-qi (Liu Zi Hu Xi Xuan Fei Qi). This posture involves inhalation and exhalation through six different mouth patterns and sounds, which are the Xu, He, Hu, Si, Chui, and Xi. This is to regulate and control the rise and fall of the breath in the body. **d** Pose 1: Xu. Put hands on waist with palms facing up. Stretch out the right arm 45° towards left front and say “Xu..”, return. Stretch out the left arm 45° towards right front and say “Xu..”, return. **e** Pose 2: He. Bend the knees. Raise the arms forward with palms up. Press palms down and say “He..”, return. **f** Pose 3: Hu. Put hands in front of abdomen, turn palms inwardly. Stretch forward with circle shape, and say “Hu..”, return. **g** Pose 4: Si. Raise hands over chest. Push hands forward with palms upright and say “Si..”, then return. **h** Pose 5: Chui. Put hands in front of the abdomen. Wrap hands around to the sides of the body and press down, say “Chui..”, then return. **i** Pose 6: Xi. Put hands in front of the abdomen, raise the palms above the head, and open arms. Pull back and press down, say “Xi..”, then return. **j** Posture 3: Expand and draw in chest to harmonize qi and blood (Kai He Kuo Xiong He Qi Xue). Cross arm over chest. Push palm towards, split apart, and open arms and turn head to the left hand side. Hold for 5 s and return. Repeat on the other side. **k** Posture 4: Push and pull hard to increase strength (Guan Jing Tui La Zeng Qi Li). Turn the toes inward. Put the hands on waist with expanded chest and extended shoulders, hold for 5 s. Push hands forward with force until strength reaches the fingertips, then say “He..”, push forward and straighten the elbows. Hold for 5 s. After inhaling, pull the hands back quickly while saying “He..”, put the hands on waist. **l** Posture 5: Fly obliquely to balance Yin and Yang (Tai Ji Xie Fei Tiao Yin Yang). Shift weight to the right, bend the knees, left empty step, hold hands in a circle, step to the left, pull the hands apart. Shift weight to the left, hold hands in a circle, step to the left, pull the hands apart. **m** Posture 6: Lift the heels and stomp the feet to help eliminate diseases (Ti Zhong Dun Zu Xiao Bai Bing). Put hands around waist, slowly lift heels off the ground. Hold for 5 s and stamp on heels, return. **n** Posture 7: Closing form (guide Qi to Dantian and regulate tendons and joints). Slightly bend the knees, cross hands over the chest. Open arms and kick left, return. Open arms and kick right, return. Cross hands over your chest, open arms outwards, return. Guide Qi to Dantian, return

Kangyi Qiangshen Gong exercises are performed as follows (Fig. 4).

1. Preparation phase
  Posture 1: Starting form and warm-up. This posture consists of three poses including Ding Tian Li Di (Stand upright to touch the sky), Tong Zi Bai Fo (Child Worships Buddha), Yang Tou Wang Yue (Looking up to the moon).
  Pose 1: Stand upright to touch the sky (Ding Tian Li Di). Lift the hands slowly, press down. Raise hands in front of the chest, with knees slightly bent. Lift hands above the head, with palms facing up, fingertips facing, while standing on your toes. Stretch upward, hold for 5 s, return to standing position.
  Pose 2: Child worships Buddha (Tong Zi Bai Fo). Arms raised from the sides of body, parallel to the floor and stretch outwards, then hold for 5 s. Press palms together, flip hands 180°, and align the fingertips towards Dan Zhong acupoint, breathe naturally, hold for 5 s.
  Pose 3: Look up the moon (Yang Tou Wang Yue). Turn toes inward, support the back of neck with the right hand, and the back with the left hand. Raise the head to the right 45° and twist waist, hold for 5 s then return. Twist waist and look at the left heel, hold for 5 s. Support the back of neck with the left hand, and the back with the right hand. Raise the head to the right 45° and twist waist, hold for 5 s then return. Twist waist and look at the right heel, hold for 5 s.
2. Practice phase
  Posture 2: Six sounds’ breathing exercise to exhale stagnated lung-qi (Liu Zi Hu Xi Xuan Fei Qi). This posture involves inhalation and exhalation through six different mouth patterns and sounds, which are the Xu, He, Hu, Si, Chui, and Xi. This is to regulate and control the rise and fall of the breath in the body.
  Pose 1: Xu. Put hands on waist with palms facing up. Stretch out the right arm 45° towards left front and say “Xu…”, return. Stretch out the left arm 45° towards right front and say “Xu…”, return.
  Pose 2: He. Bend the knees. Raise the arms forward with palms up. Press palms down and say “He…”, return.
  Pose 3: Hu. Put hands in front of abdomen, turn palms inwardly. Stretch forward with circle shape, and say “Hu…”, return.
  Pose 4: Si. Raise hands over chest. Push hands forward with palms upright and say “Si…”, then return.
  Pose5: Chui. Put hands in front of the abdomen. Wrap hands around to the sides of the body and press down, say “Chui…”, then return.
  Pose 6: Xi. Put hands in front of the abdomen, raise the palms above the head and open arms. Pull back and press down, say “Xi…”, then return.
  Posture 3: Expand and draw in chest to harmonize qi and blood (Kai He Kuo Xiong He Qi Xue). Cross arm over chest. Push palm towards, split apart and open arms and turn head to the left side. Hold for 5 s and return. Repeat on the other side.
  Posture 4: Push and pull hard to increase strength (Guan Jing Tui La Zeng Qi Li). Turn the toes inward. Put the hands on the waist with expanded chest and extended shoulders, hold for 5 s. Push hands forward with force until strength reaches the fingertips, then say “He…”, push forward and straighten the elbows. Hold for 5 s. After inhaling, pull the hands back quickly while saying “He…”, put the hands on waist.
  Posture 5: Fly obliquely to balance Yin and Yang (Tai Ji Xie Fei Tiao Yin Yang). Shift weight to the right, bend the knees, left empty step, hold hands in a circle, step to the left, pull the hands apart. Shift weight to the left, hold hands in a circle, step to the left, pull the hands apart.
  Posture 6: Lift the heels and stomp the feet to help eliminate diseases (Ti Zhong Dun Zu Xiao Bai Bing). Put hands around waist, slowly lift heels off the ground. Hold for 5 s and stamp on heels, return.
  Posture 7: Closing form (guide Qi to Dantian and regulate tendons and joints). Slightly bend the knees, cross hands over the chest. Open arms and kick left, return. Open arms and kick right, return. Cross hands over your chest, open arms outwards, return. Guide Qi to Dantian, return.

### Adherence

This will be home respiratory training program with compliance assessed every week by telephone calls and review of the everyday charts filled-in by participants. The participants will be informed to perform breathing techniques or KQG exercises every day at 10 am and 4 pm. Remote monitoring devices will be offered to every ward so that remote monitoring of the training of the subject can be achieved. The participants will be required to sign in a recorded diary after each exercise.

### Outcome measurements

Researchers assessing primary outcomes were blinded for group assignment. The outcomes include Modified Medical Research Council Dyspnea Scale, Modified Borg Scale, Fatigue Scale-14, Patient Health Questionnaire-9 Scale, Pittsburgh Sleep Quality Index and Respiratory Symptoms Scale. Evaluation of the outcomes will occur at three points (pre-exercise, 3 months post-exercise, and 3 months follow-up).

### Primary outcome measurements

#### Modified Medical Research Council (mMRC) Dyspnea Scale

The mMRC Dyspnea Scale will be used as the primary outcome indicator to evaluate the dyspnea symptoms and physical health; mMRC score is a good indicator of the functional capacity of patients’ lungs [30]. The mMRC grades [31] are as follows: mMRC grade 0, dyspnea occurs only during strenuous exercise; mMRC grade 1—the patient has shortness of breath when walking on flat ground or walking on a small slope; mMRC grade 2—due to shortness of breath, when walking on flat ground, the patient is slower than another person of the same age or needs to stop to rest; mMRC grade 3—when walking on flat ground for approximately 100 m or after a few minutes, the patient needs to stop to pant; and mMRC grade 4—due to severe breathing difficulties, the patient cannot leave the house or has breathing difficulties when dressing or undressing. The mMRC score will be assessed with a repeated longitudinal analysis.

### Secondary outcome measurements

#### Modified Borg Scale

The Modified Borg Scale [32] will be used to assess the dyspnea symptoms and physical health. The participants will be required to walk for 6 min in a corridor with the distance of 30 m and to fill in the questionnaire or to describe verbally of the degree of dyspnea. The Modified Borg score grades are as follows: grade 0—no breathing difficulties; grade 1—very mild breathing difficulties or fatigue; grade 2—mild breathing difficulties or fatigue; grade 3—moderate breathing difficulties; grade 4—slightly serious breathing difficulties; grade 5—relatively serious breathing difficulties; grade 6—degree of breathing difficulties is between grades 5 and 7; grade 7—very severe breathing difficulties; grade 8—degree of breathing difficulties is between grades 7 and 9; grade 9—extremely difficult in breathing; grade 10—maximum degree of breathing difficulties (patient have extreme feeling of breathing difficulties, it is the most severe breathing difficulties that he/she has ever experienced). After all the patients finished the Borg Scale, the grade is classified into grade 0, grades 1 to 3, grades 4 to 6, and grade ≥ 7. All patients and their family dependent will sign the consent letter [33].

#### Fatigue Scale-14

The aim of Fatigue Scale-14 is to investigate the subjective feelings in fatigue of the patients. The patients will choose “yes” or “no” based on the situation that suits them the most. The Fatigue Scale-14 consists of 14 questions, in which questions 1–8 are physical fatigue while questions 9–14 are mental fatigue. Among the 14 questions, 11 questions are positive scoring, which “yes” is scored as 1 mark, “no” is scored as 0 mark; 3 questions, which are questions 10, 13, and 14 are negative scoring, which “no” is scored 1 mark and “yes” is scored as 0 mark. The physical fatigue is calculated based on questions 1–8 and its maximum score is 8; the mental fatigue is calculated based on questions 9–14 and its maximum score is 6. The Fatigue Scale-14 is the sum of the physical fatigue and the mental fatigue, the maximum score is 14. The greater the value of the Fatigue Scale-14, the greater the fatigue degree [34].

#### Patient Health Questionnaire-9 Scale (PHQ-9)

The Patient Health Questionnaire-9 Scale is used to measure the patients’ depression and seriousness in medical populations clinically; the Patient Health Questionnaire-9 Scale has been carried out with the usage of medical populations in various setting. The Patient Health Questionnaire-9 Scale is used to assess the frequency of the 9 negative objects that occurred to the patients in the past fortnight. It is scored as 0 point if it is not at all; it is scored as 1 point if they occurred once every few days; it is scored as 2 points if they occurred once more than half a day; it is scored as 3 points if they occurred every day. The Patient Health Questionnaire-9 Scale is classified into categories in increasing seriousness which include 0–4, 5–9, 10–14, 15–19, and ≥ 20. A repeated longitudinal analysis will be used to evaluate the outcome of the Patient Health Questionnaire-9 Scale [35].

#### Pittsburgh Sleep Quality Index (PSQI)

The Pittsburgh Sleep Quality Index (PSQI) assesses sleep quality and disturbances over the duration of experiment [36, 37]. Nineteen individual items generate seven “component” scores: subjective sleep quality, sleep latency, sleep duration, habitual sleep efficiency, sleep disturbances, use of sleeping medication, and daytime dysfunction. The total score of these seven components is the global score.

#### Respiratory Symptoms (RS) Scale

Respiratory symptoms include cough, expectoration, fatigue, chest tightness, dyspnea, sore throat, nasal congestion, runny nose, and some other nonspecific symptoms [38, 39]. The severity of these symptoms will be recorded accordingly as 0–10, with 0 being no respiratory symptom and 10 being the most severe. A repeated longitudinal analysis will be used to evaluate these self-evaluation scales.

### Sample size calculation

The PASS software (PASS 11, NCSS, LLC, Kaysville, UT, USA) will be used to estimate the sample size utilizing two independent sample means (*α* = 0.05, *β* = 0.10). We will use the mMRC Scale as the primary efficacy outcome. Based on previous clinical studies on the effect of respiratory symptoms after KQG interventions, the mMRC Scale score in the control group is 0.74 with a standard deviation of 0.55, and the average mMRC Scale score in the treatment group is 1.52 with a standard deviation of 0.54 [25, 40, 41]. The target sample size will be 60 participants in each group based on 80% power, 5% type I error, and two-sided test, anticipating on maximum loss to follow-up of 20%.

### Randomization

After the baseline measures, we will use a random number generator (IBM, Chicago, IL, USA) to randomly assign eligible participants to either BT group or KQG group and the randomization sequence will be sealed into the sequenced opaque envelope. The participants who pass the screening will then receive the envelope. The Science and Technology Department, Acupuncture & Tuina Institute SHUTCM will be responsible for the randomization.

### Blinding

We will use a concealed randomization where neither participant nor researcher was aware of the randomization until after the baseline interview. None of the participants or clinicians will be blinded to treatment allocation after randomization, due to the specific exercise intervention. However, they will be blinded to the outcome assessment. Evaluation of the outcomes will be accomplished by independent evaluators (IEs), including the outcome assessors, the data managers, and the statisticians. IEs will be blinded until the clinical database is locked.

### Data collecting and monitoring

Data monitoring will be conducted by the Science and Technology Department of Acupuncture &Tuina Institute, SHUTCM. Clinical test data will be captured through case report forms (CRFs) by paper questionnaires or by online data capture applications and store in a secured location. Confidentiality will be maintained through the assignment of an encrypted number code. Data entry was double-checked for accuracy. The test data will be locked and analyzed by two statisticians under the supervision of a third statistician and the administrators. Then, the test data will be recorded on the sub-website of China Clinical Trial Center (http://www.medresman.org.cn/login.aspx) electronic data management system. The electronic database will be closed after data entry is completed. The administrators are required to report weekly data monitoring progress to the Steering Committee regularly, including the accuracy and reliability of data.

Trial auditing involves periodic independent review of core trial processes and documents, such as data monitoring and scheduling monitoring. Data monitoring will be conducted by the Science and Technology Department of Acupuncture &Tuina Institute, SHUTCM. The monitors will verify that all adverse events were documented in the correct format and are consistent with protocol definition. The monitor will verify the following variables for all patients: initials, date of birth, sex, signed informed consent, eligibility criteria, date of randomization, treatment assignment, adverse events, and endpoints. Scheduling monitoring visits will be a function of COVID-19 patient enrollment, site status, and other commitments. The investigators must be available to meet with the monitors. If a problem is identified during the visit (i.e., inadequate or insufficient staff to conduct the study, missing study documents), the monitor will assist the site in resolving the issues. The focus of the visit/electronic monitoring will be on source document review and confirmation of adverse events.

### Statistical analysis

#### General analysis principles

The statistical analysis plan of this trial refers to the previous published study protocol [25, 27]. In the case of continuous variables, Lilliefors corrected Kolmogorov–Smirnov tests will be applied to assess the normality. Continuous variables will be presented as descriptive statistics (mean ± the standard deviation (SD) for data with normal distribution or median (interquartile range) for nonnormally distributed data). Categorical data will be calculated with frequency counts and percentages of participants. Tests of statistical significance will be conducted at the two-tailed *α* level of 0.05. Statistical analysis will be performed in IBM SPSS version 28 (IBM Corp., Armonk, NY, USA).

#### Analysis of primary outcome

The primary outcomes will be evaluated at baseline, 12 weeks after the intervention, and follow-up. We will conduct sensitivity analyses for missing data (intention-to-treat analysis) and for adjustment of covariates of physical activity (adjusted intention-to-treat analysis). The primary endpoint is the mean change in mMRC Scales from baseline to the follow-up day. Baseline covariates included are sex, age category, history of cardiopulmonary disease, and body weight category. The whole outcomes scale will be measured at baseline. When normally distributed, the data will be analyzed with unpaired *t*-test for comparison between two groups, while for multiple comparisons one-way ANOVA and repeated measures (RM) ANOVA will be used. A sensitivity analysis using multiple imputation techniques (imputation using chained equations) will be included to assess the effect of missing data on the primary outcome. Besides, we will compare participants randomly allocated to BT group or KQG group, who received an allocated treatment that was deemed to be of high fidelity. The percentage of missing data is 20% and 5 imputed datasets will be used for analysis, which is the minimum recommended number. Prior to any analysis, any missing data pattern will be investigated and the reasons for missing data will be obtained and patterns will be investigated to judge the plausibility of missingness assumptions.

#### Analysis of secondary outcome

Analysis of secondary outcome will be undertaken referring to analysis of primary outcome. We will test for between arm differences for each endpoint specified. Chi-square test or Fisher’s exact test will be adopted to analyze the adverse events (AEs).

### Quality control

The study will be overseen by a steering committee during the whole processing of the trial. All the researchers will be trained with the trial methodology and monitoring technique before participating in the trial. Participants will be remotely monitored online and supervised offline by coordinators.

### Safety evaluation

Safety evaluations will be conducted at screening, throughout the trial, and at follow-up 12 weeks after the last TCMRE, although TCMRE programs are low risk. Any adverse events (AEs) will be collated from CRFs and follow-up questionnaires. Even though BT and KQG are at low risk. AEs will be collated from CRFs and follow-up questionnaires. Researchers will then analyze whether AEs are directly related to the rehabilitation program. When sports injuries occur or condition suddenly deteriorates during the trial with serious complications or severe adverse effects such as breathing difficulties, severe anxiety, severe depression, the trial will be terminated immediately and prompt medical treatment will be administered according to the condition of the subject. Researchers will then analyze whether they are directly related to the rehabilitation program.

## Discussion

In March 2022, a large-scale outbreak of coronavirus disease occurred again in Shanghai, China. As the number of discharged patients increased, the sequelae of discharged patients gradually became the focus of clinical attention [2]. Despite the relatively low overall mortality rate as compared to the previous COVID-19, yet fatigue, depression, or cognitive impairment (brain fog) symptoms encountered by discharged patients also need immediate and appropriate rehabilitation treatment. The prevalence of sleep problems during the COVID-19 pandemic is approximately 40% of people from the general and health care populations [42]. The Chinese government has taken instant medical measures and rescue efforts to control further deterioration of the pandemic. These measures include intensive surveillance, epidemiological surveillance, symptomatic supportive treatment, and active application of Traditional Chinese medicine (TCM), for the purpose of reducing mortality and shortening hospital stay [25, 40]. All medical expenses for COVID-19 disease treatment are fully subsidized by the China Health Insurance; however, the convalescence treatment of discharged patients is still a huge medical burden. Therefore, it is necessary to find more non-drug therapies to cope with the sequelae of this pneumonia.

Taiji [43, 44], Yijinjing Qigong [45], Shaolin Neigong [46], Wuqinxi Qigong [47], Liuzijue Qigong[48], and Baduanjin Qigong [49, 50] belong to the category of supplementary medicine and alternative medicine and are widely used in disease prevention, treatment, and rehabilitation [40, 51, 52]. Some researchers [52–55] have attested that Taiji, Yijinjing Qigong, Shaolin Neigong, Wuqinxi Qigong, Liuzijue Qigong, and Baduanjin Qigong can improve the clinical symptoms, along with physical and mental health of patients with lung diseases. TCMRE projects are highly operational and cost-effective due to overburdened healthcare system [56, 57]. Studies found that exercise may be beneficial for improving pulmonary symptoms and suppressing inflammation in discharged patients [49]. KQG exercise prescription adopted in this study consists of seven exercises: Starting form and warm-up, Liu Zi Hu Xi Xuan Fei Qi, Kai He Kuo Xiong He Qi Xue, Guan Jing Tui La Zeng Qi Li, Tai Chi Fei Tiao Yin Yang, Ti Zhong Dun Zu Xiao Bai Bin and losing form. KQG is a low-risk therapy that combines the advantages of Taiji, Yijinjing Qigong, Shaolin Neigong, Wuqinxi Qigong, Liuzijue Qigong, and Baduanjin Qigong, with neither need for heavy equipment nor constrained by time or location [17]. KQG is more targeted to the cardiopulmonary function, fatigue state, and emotional disturbance of COVID-19 patients. By combining literature research and the actual application of KQG exercise in Wuhan Leishenshan Hospital and Huangshi Hospital of Traditional Chinese Medicine, KQG is considered eligible to participate in the rehabilitation treatment of discharged COVID-19 patients [17, 25]. KQG will promote recovery for the majority of patients and improves the living quality of patients [17, 25]. The human immune system is able to counteract the damage of foreign microorganisms through dynamic regulation of innate immunity by TCMRE [40, 57], and increasing muscle strength and respiratory muscle endurance to relieve dyspnea, anxieties, and tension associated with negative situations [25]. TCMRE is capable of coordinating Qi and blood, adjusting the balance of Yin and Yang from the macro theory of TCM. Nonetheless, stronger evidence is needed to further demonstrate the effectiveness of KQG exercise prescription in improving clinical symptoms of COVID-19 discharged patients. Thus, this clinical trial will further evaluate the rehabilitation effect of KQG on discharged patients.

## Study limitations

An inevitable limitation of this study is that BT intervention and KQG intervention cannot blind participants and therapists. The outcomes may be affected because patients can know their group and anticipate the treatment effect in advance.

## Trial status

This trial will recruit patients on May 2022 and is expected to end in December 2022. This protocol is v.10 (the 1st version) dated April 2022.

## Data Availability

The protocol manuscript does not contain any data, so we declare that data and materials are not applicable in this study. However, the datasets generated and analyzed during the study will be available in the [Figshare] repository when the trial is complete.

## Abbreviations

COVID-19: 2019 Novel coronavirus
TCMRE: Traditional Chinese medicine rehabilitation exercises
KQG: Kangyi Qiangshen Gong exercise prescription
BT: Breathing techniques according to WHO
RCT: Randomized controlled trial
SPIRIT: The Standard Protocol Items: Recommendations for Interventional Trials
LF: The principal investigators
WC: Study coordinator
mMRC: Modified Medical Research Council
PSQI: Pittsburgh Sleep Quality Index
